# A rare case of giant leiomyosarcoma in a filarial scrotum: a case report

**DOI:** 10.1186/1477-7819-9-20

**Published:** 2011-02-10

**Authors:** Majid Ahmed Talikoti, SV S Deo, Nootan K Shukla, Ashwin A Kallianpur, Mamraj Gupta

**Affiliations:** 1 Department of Surgical Oncology, BRAIRCH, AIIMS, New Delhi, 110029, India

## Abstract

Giant leiomyosarcoma of scrotum is a rare tumour. A case of scrotum leiomyosarcoma is presented in a 67 year old patient with scrotal filariasis which was managed successfully with total scrotectomy with bilateral orchidectomy, degloved penis reconstructed with rotation advancement supra pubic fasciocutaneous flap. We made a literature search proving the rarity of this lesion type. Only 36 cases have been described and the first case in a filarial scrotum

## Introduction

Leiomyosarcoma of the scrotum is a rare tumour. Scrotal leiomyosarcomas (LMS) are slow growing tumours that present as firm rubbery nontender irregular mass [1]. They may arise from paratesticular or scrotal skin. Over 95% of all paratesticular leiomyosarcomas are located in the spermatic cord or epididymis; their location in the scrotal skin is exceptional. To date approximate 36 scrotal LMS have been reported in literature. We report a rare case of giant primary scrotal LMS arising in a filarial scrotum. There is no report of such giant LMS and none in the background of scrotal elephantiasis.

## Case Presentation

A 67 years old patient of high socioeconomic status reported to the outpatient clinic with complain of rapidly ulcerated mass in the scrotum. He had history of filariasis and scrotal swelling of more than 40 years duration. His Eastern Cooperative Oncology Group (ECOG) performance Status was 3, capable of only limited self care, confined to bed or chair more than 50% of waking hours, due to large painful swelling of the scrotum [2].

**Figure 1:**
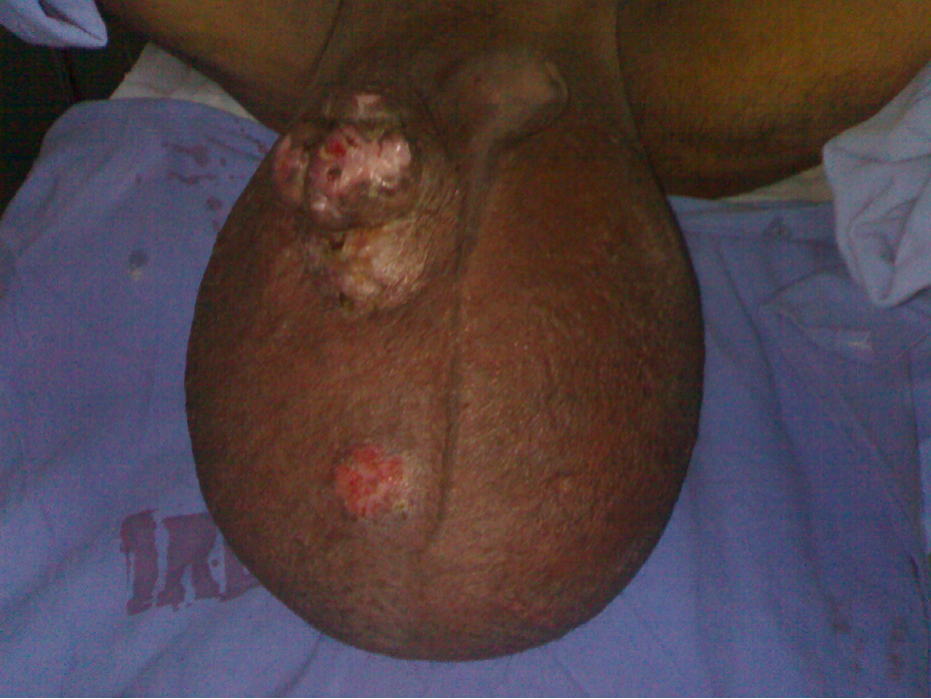
Large 30x30 cm tumour with overlying ulcer over the right scrotum.

**Figure 2:**
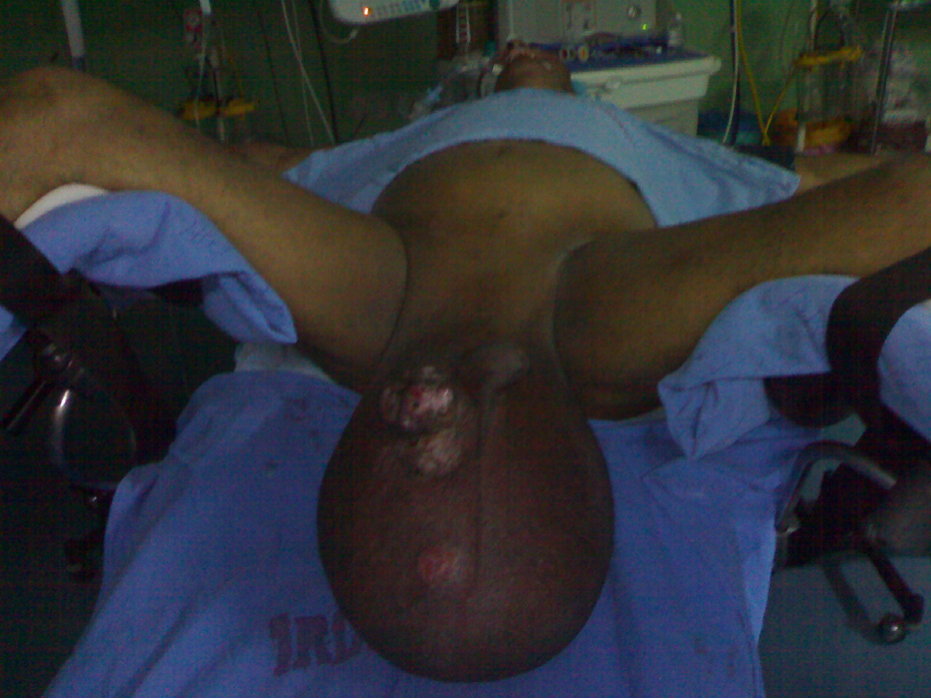
Large 30x30 cm tumour with overlying ulcer over the right scrotum.

**Figure 3:**
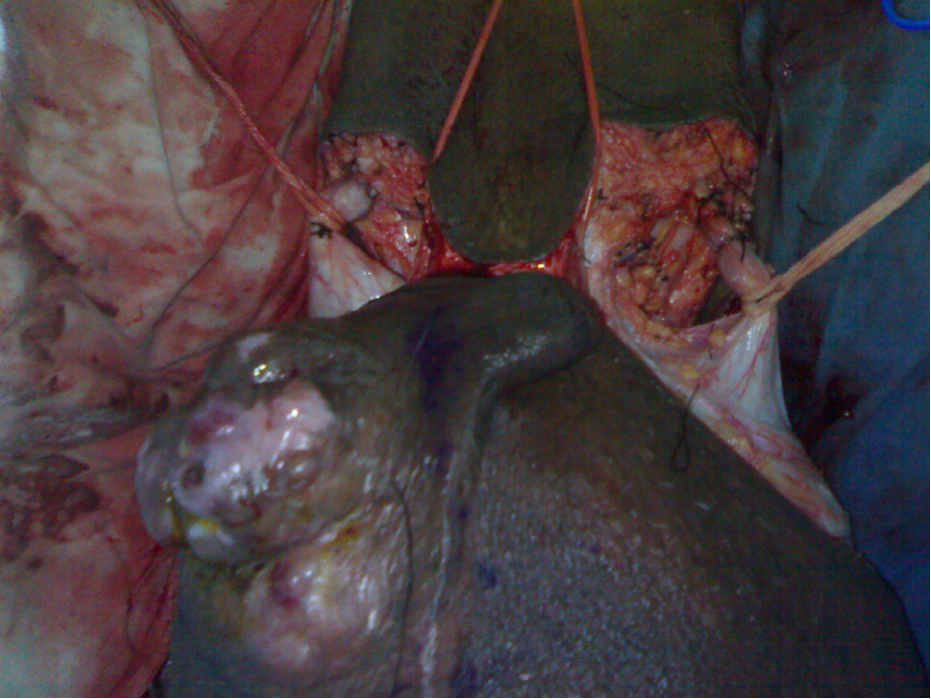
Radical resection of tumour.

**Figure 4:**
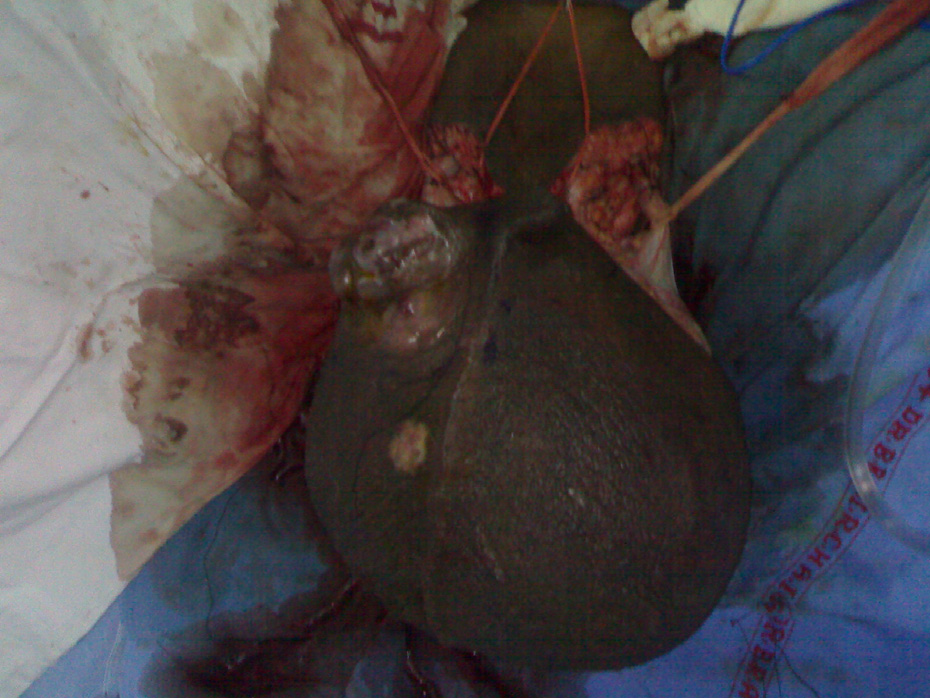
Radical resection of tumour.

**Figure 5:**
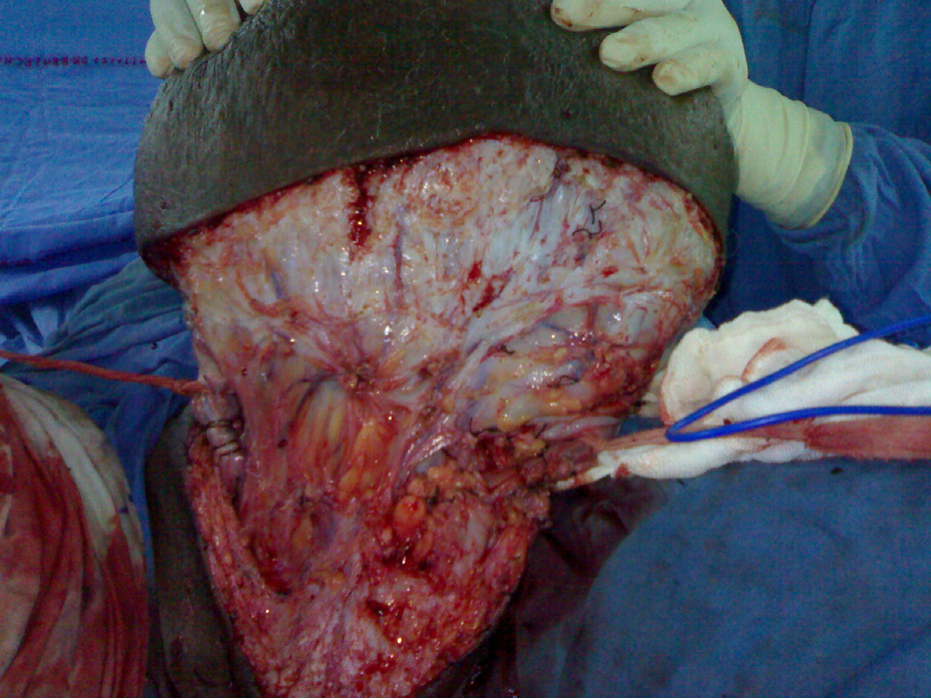
Radical resection of tumour.

**Figure 6:**
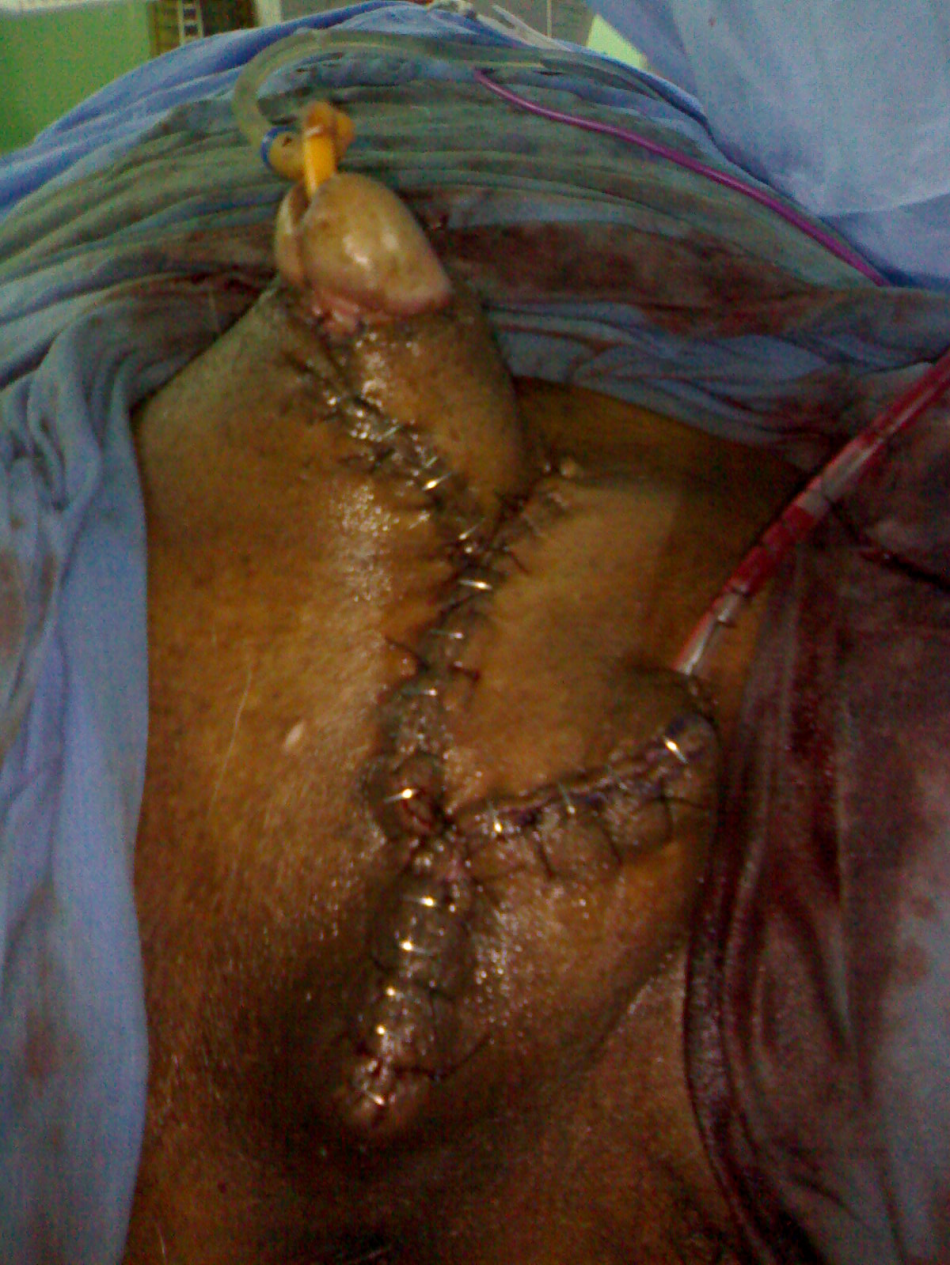
Reconstruction of degloved penis done with suprapubic fasciocutaneous flap.

On local examination, huge filarial scrotum with skin changes and buried penis, 30 × 30 cm ulcerated firm diffuse mass of the scrotal skin encroaching over the root of penile skin [figures 1, 2]. The tumour was mobile not fixed to the testes. Metastatic work up included CT chest and abdomen and was negative. Wedge biopsy was compatible with Leiomyosarcoma. After an informed written consent patient underwent a radical resection of tumour including total scrotectomy and bilateral orchidectomy, degloving of buried penis with reconstruction by rotation advancement supra pubic fasciocutaneous flap [figures 3, 4, 5, 6]. Patient recovery was uneventful. Patients ECOG performance scale improved from 3 to 1, he became ambulatory with his ability to perform routine plus outdoor activity on his own.

Post operative histopathology revealed 28 × 25 × 15 cm fleshy tumour with an over lying ulcer. Testes were not involved by the tumour. All resected margins were negative. Microscopically a malignant mesenchymal neoplasm was identified with smooth muscle differentiated intermingling bundle of cells with eosinophilic abundant mitotic figure 15 mitosis per 10 high power. Tumour was positive for actin, vimentin and desmin and negative for S 100. Based on histopathology findings and Immunohistochemistry markers diagnosis of scrotal leiomyosarcoma was made.

Patient received postoperative external beam radiation 60G/30fractions. He is on regular follow up for past 19 months and is disease free.

## Discussion

Soft tissue Sarcomas are 1% of all malignancies. Leiomyosarcomas constitutes 10 to 20% of soft tissue sarcomas. They arise most often from uterus, gastrointestinal tract and retroperitoneal region [1]. Subcutaneous LMS are 1 to 2% of all superficial soft tissue malignancies [3]. Among genitourinary sarcomas in adults, leiomyosarcomas are the most common type and arises in the bladder, kidney, or prostate. Its origin in scrotum is exceptional with only 36 cases have been reported in literature. LMS are malignant mesenchymal line neoplasm with size of the tumor varying from 2 to 9 cm, with an average of 5 cm. Biopsy is done to confirm the diagnoses of LMS. A confirmative diagnosis of LMS is based on histological examination. They show spindle cells with cigar shaped nuclei arranged in interweaving fascicles [1]. On Immunohistochemistry they are positive for actin, desmin and CD 34 [4]. The mode of spread is primarily haematogenous to lung, liver, and bone. Prognoses of LMS depend on tumour size, depth, grade and evidence of distant metastases.

Scrotal elephantiasis is caused by acquired filarial infestation with Wuchereria bancrofti. Occasionally it has been attributed to radiotherapy, neoplasm and lymphadenectomy. It is emotionally distressing and physically disabling. With problems of hygiene, urinary incontinence, unesthetic appearance, loss of libido and immobility are severely debilitating symptom [5].

Filariasis in advance stages may evolve into scrotal lymphedema and scrotal elephantiasis. In 1948 Stewart and Treves first described the association of chronic lymphedema with lymphangiosarcoma [6]. Lymphangiosarcomas are common malignancies in those with chronic filarial infections [7]. Sarcomas have been seen after filarial infection and chronic lymphedema [8]. Scrotal leiomyosarcoma have a potential of distant metastases. Here in we report the first case of large Leiomyosarcoma of a filarial scrotum. Scrotal Leiomyosarcoma in our case was a high grade, large size, stage III having a potential of distant metastases.

The paucity of literature in this area often makes treatment decisions difficult. Simple excision proved to be inadequate treatment for sarcomas in the paratesticular region. In the Princess Margaret Hospital report, wide excision revealed microscopic residual disease in 27% of completely excised cases [9]. The primary treatment of LMS of scrotum is complete resection with histological negative margins. The difficulty in achieving an oncological safe margin reflects the tumour biology. An aggressive initial resection is required at the time of the first operation [10]. Many surgical methods have been described for scrotal and penile reconstruction. Like pedicle groin flap based on superficial circumflex iliac artery to cover the penis and bilateral superior medial thigh flap for scrotal reconstruction [11]. We preferred a single staged, simpler reconstruction by using rotation advancement flap of the supra pubic area over the two staged pedicle groin flap to reconstruct the penis. The scrotal defect was primarily closed. For most patients, local control is improved with preoperative or postoperative radiotherapy. Hensley and colleagues were the first to report the activity of the gemcitabine-docetaxel combination in patients with leiomyosarcomas [12]. The role of chemotherapy for high-risk patients remains controversial, but chemotherapy is used at several major centres' for high-risk patients. In our case we treated our patient with wide local excision and postoperative 60 Gy radiotherapy.

## Conclusions

• Scrotal leiomyosarcoma is a rare clinical entity and scrotal leiomyosarcoma in a filarial lymphadenomatous scrotum; this is the first case to be reported.

• Aggressive surgical resection including tumour and diseased filarial skin is recommended.

## Consent

Written informed consent was obtained from the patient for publication of this case report and accompanying images. A copy of the written consent is available for review by the Editor-in-Chief of this journal.

## Competing interests

The authors declare that they have no competing interests.

## Authors' contributions

MT, SVSD, NKS, AK and MG prepared the manuscript and reviewd the literature. All authors read and approved the final manuscript.
